# Prof. Shuchün Teng: a paragon taxonomist of great passion and firm belief

**DOI:** 10.1007/s13238-016-0348-4

**Published:** 2016-12-01

**Authors:** Guojie Li

**Affiliations:** State Key Laboratory of Mycology, Institute of Microbiology, Chinese Academy of Sciences, Beijing, 100101 China

Shuchün Teng (or Shu-Ch’un Teng, 1902–1970, Fig. 1), courtesy name Zimu (子牧), was one of the most famous Chinese mycologists. His main achievements were in fungal taxonomy, plant pathology and forest ecology. Being the author of “Fungi of China”, the first monograph of fungi in China, he was regarded as “a paragon taxonomist” and “truly an immerse taxonomic legacy in which all China and all Chinese people can find pride”. In addition to being a studious mycologist in pursuit of truth and honesty all his lifetime, his diligence, thoroughness, optimism, and patriotism also made him a conscientious leader, a respected mentor, a brave mycology and forestry pioneer and a good father (Cheng; Deng et al. 2012; Korf 2002).

**Figure 1:**
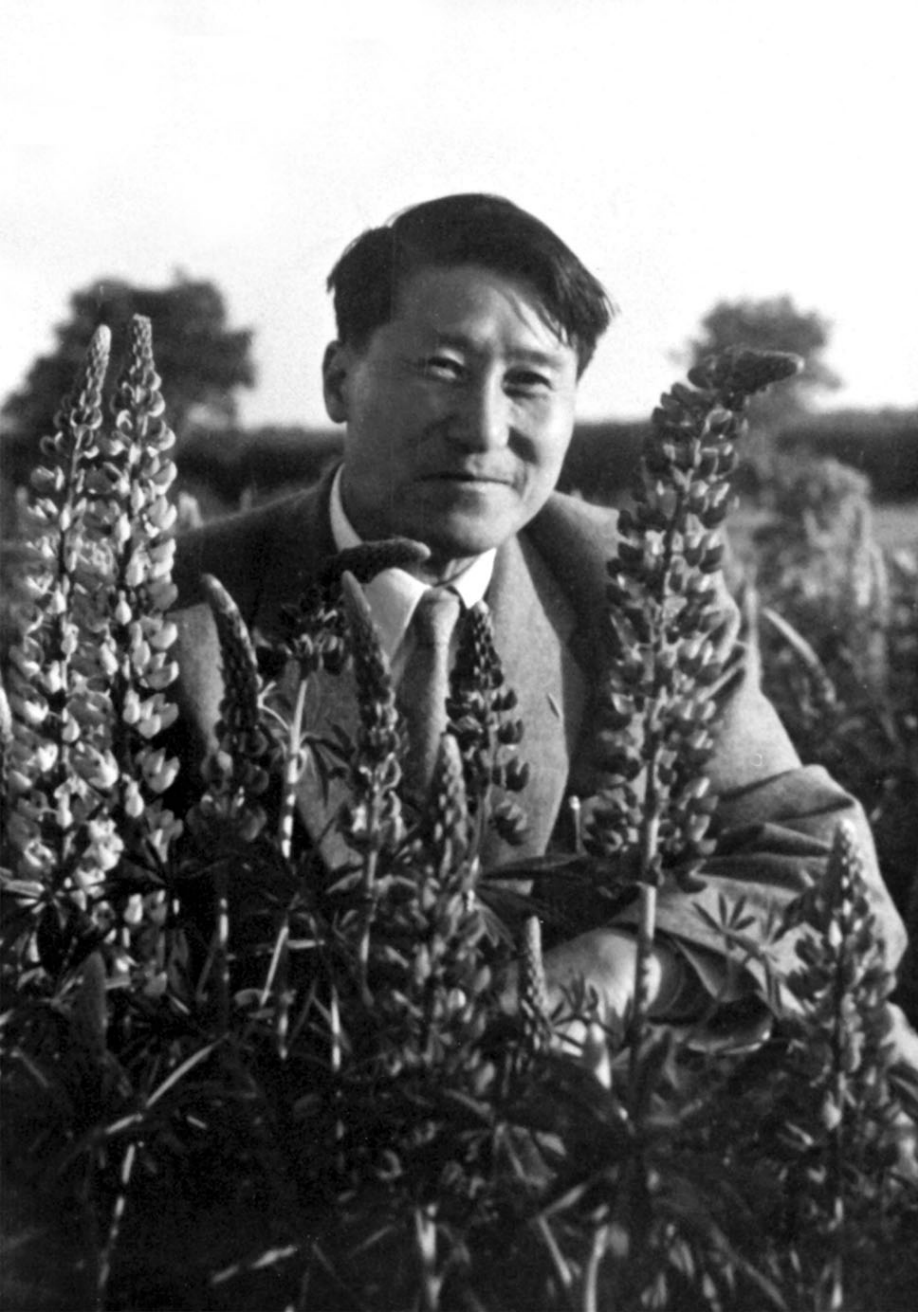
Prof. Shu-Chün Teng (1902–1970)

Born and raised in a large and needy family in Min County (currently known as Minhou, Foochow or Fuzhou), China, Shuchün Teng’s parents are both teachers. He received early childhood elementary education in primary school of Minhou. By taking part in the entrance examination of Tsing Hua College, he got the admission qualification of Tsing Hua in 1914. He spent 6 years there as a student of preparatory course class for the Boxer Indemnity Scholarship Program (Cheng).

**Figure 2:**
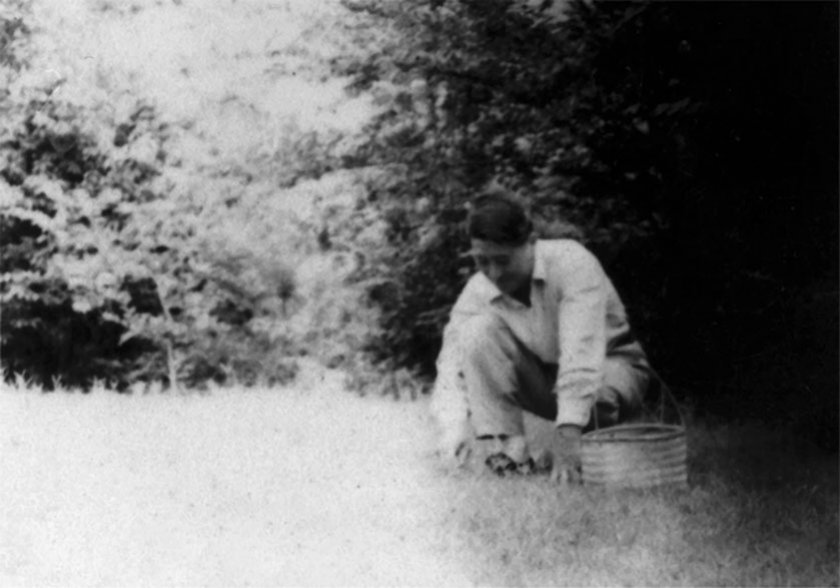
Prof. Shu-Chün Teng in field work

In 1923, Shuchün Teng entered the Department of Forestry, College of Agriculture in Cornell University, and got his bachelor’s degree in Agriculture and master’s degree in Forestry within 3 years. During that time, he also worked as a part-time farmer and carpenter in suburb farms. When taking his Ph.D. courses on phytopathology, he won two gold key badges for Phi Kappa Phi and Sigma Xi, respectively, and was invited as the member of the two honor societies of science. With these honors, he went back to China in 1928, even before the completion of his Ph.D. The most important thing at that time was getting ready to work on the mycology in China, so the granting of the doctoral degree was as light as a feather for him (Cheng) (Fig. 2).

**Figure 3:**
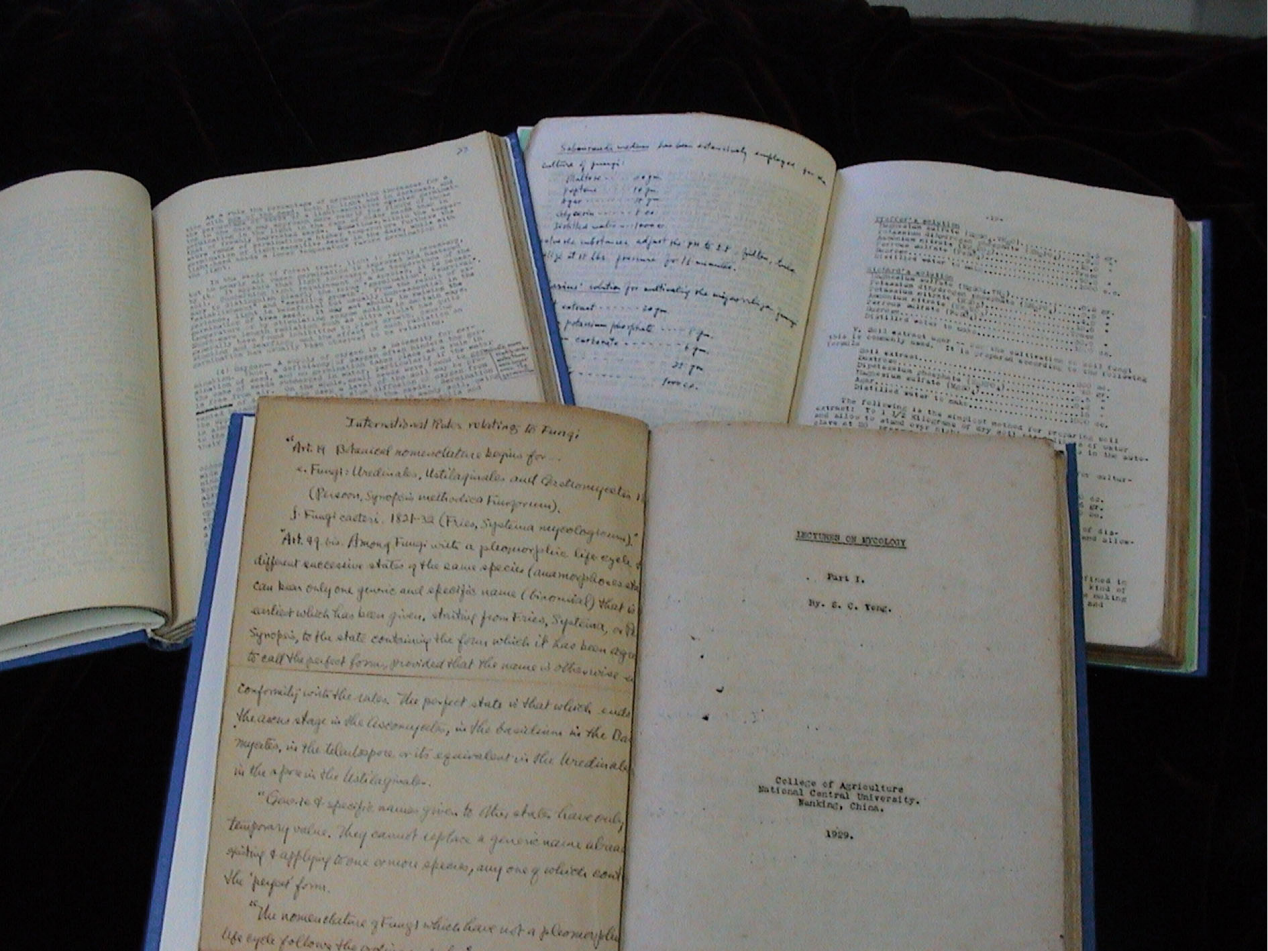
The manuscript of Prof. Shu-Chün Teng

From 1928 to 1931, Shuchün Teng was professor in Lingnan University, Private University of Nanking, and National Central University. He mainly worked on the disease control and treatment of rice, wheat and cotton, and in the meantime, he also taught mycology and plant pathology. From 1932 to 1941, he was appointed as the professor in Institute of Biology, Science Society of China, Central History and Nature Museum, Institute of Botany, Academia Sinica, and Institute of Forestry Experiment, Academia Sinica. China has a variety of forestry and fungal resources, because of the large territory which covers several climate zones and, but the investigations of such resources at that time were almost blank. During the first ten year after his returning to homeland (1928–1938), he identified 1400 taxa of 400 fungal genera based on specimens collected by himself, and published 34 research articles, in which 5 new genera and 121 new species were described (Fig. 3). In 1939, his first monograph “A contribution to our knowledge of the higher fungi of China” was published. This book covered the morphology, hosts, habits and habitats information about 1391 species of 23 orders, 75 families, and 387 genera in Basidiomycota, Ascomycota and Deuteromycota (*viz*. imperfect fungi).

From the early years of 1930s, Shuchün Teng switched his attention to forestry ecology. He organized a survey team with the grant support from the Ministry of Agriculture and Forestry. In 1939, he and his 12 young colleagues spent several months on investigating the virgin forests of Sichuan (四川), Yunnan (云南) and Xikang (西康) Provinces in southwestern China (Fig. 2). They clarified the component, distribution, cumulation, diseases and insect pests of the forest in these areas. As the invasion of Japanese went on in 1940, Shuchün Teng and his colleagues packed up and sent over 2000 Chinese specimens to the US *via* Indochina Peninsula by ox-cart, train and ship. Although the specimens he collected remained in China were almost destroyed during that time, the duplicates kept in US are still in good conditions today, 2278 specimens of which were returned to China in 2009. In 1941, Prof. Teng declined the deputy minister of Ministry of Agriculture and Forestry, and moved to Jone (卓尼), Gansu Province, the central area of the Taohe River (洮河) forest with his family. At that time, full of danger, the upstream drainage of Taohe River was a remote, wild, inaccessible area. He travelled over the hills and through the rivers, suffered the rigors of living in the wilderness, and kept his gun in hand all the time. In the harsh natural environment, his face was severely injured one time, and the flooded Taohe River took away his nine-year-old third daughter forever (Deng et al., 2012). Despite all of the bitterness and hardships, he never shrank back. In one of his optimistic poems, it reads “Let your hot boiling blood keep you aglow”. He proposed a complete forestry management strategy to ensure the forest construction, and its regeneration greater than the amount of cutting. He brought forward the ecological balance theory of forest in order to keep the local ecological system beneficial to forestry agriculture, farming and animal husbandry, and to decrease the soil erosion caused by flood. His efforts on ecological forestry are good samples for his successors in Gansu Province, and he was regarded as “a commemorable milestone” (Cheng).

Shuchün Teng went back to Nanjing in 1946 after the winning of Anti-Japanese War. He rebuilt the lab of fungi and established a lab for forest ecology in Institute of Botany, Academia Sinica. The data about forest and fungi that he obtained and collected in Gansu Province were reorganized, summarized and published. These publications were mainly about the forest ecology, forestation and natural forest management, forest management of eastern Qinghai-Tibetan Plateau, and forest geography in China. In 1948, he was elected as academician of Academia Sinica. He was temporarily appointed as dean of the Shenyang Agriculture College and vice chancellor of Northeast Agriculture College from 1950–1955. He dedicated all of his time and energy to the construction of the two new colleges, such as making decisions on the campus location and planning, reviewing the capital blueprint, ordering experiment equipment, etc (Cheng).

In 1955, Shuchün Teng was reassigned as deputy director of Institute of Applied Mycology, Chinese Academy of Science and the dean of the Mycology Lab. He was appointed as academic committee member (known as academicians), Chinese Academy of Science in the same year. Despite his fully filled work schedule in administration, he held on to his own fungal research at countless nights until dawns and in holidays. In order to help the young people to improve their mycological knowledge, he translated and renewed his monography (published in 1939) in Chinese version, 400 plates including Phycomycetes and slime mold taxa were added, and the total number of the species in this book entitled “Fungi of China” reached up to 2400 (Cheng). He was the founder of Herbarium of Mycology, Institute of Microbiology, Chinese Academy of Sciences (HMAS), which is the largest collection of fungal specimens in Asia at present. The basal management of the herbarium, including exsiccatae cabinet design, accession code arrangement, index system and regulations for specimen loan, all drafted by him have been retained up to today (Zheng 2002). According to the abundant sources of fungi in southern China, he also established the Central South Lab of Fungi, Chinese Academy of Sciences, which is currently known as Guangdong Institute of Microbiology, the mycological research center in South China.

Although many of his research findings are still not published, the specimen collections and publications of Shuchün Teng are regarded as the backbone for almost all the current fungal taxonomy work in China. His contribution to the development of the mycology in China is commemorated forever.
